# Individual and contextual factors influencing patient attrition from antiretroviral therapy care in an urban community of Lusaka, Zambia

**DOI:** 10.7448/IAS.15.3.17366

**Published:** 2012-06-14

**Authors:** Maurice Musheke, Virginia Bond, Sonja Merten

**Affiliations:** 1 Zambia AIDS-related TB Research Project, University of Zambia, Lusaka, Zambia; 2 Department of Epidemiology and Public Health, Swiss Tropical and Public Health Institute, Socinstrasse 57, Basel, Switzerland; 3 Faculty of Natural Sciences, University of Basel, Petersplatz, Basel, Switzerland; 4 Department of Global Health and Development, London School of Hygiene and Tropical Medicine, London, UK

**Keywords:** PLHIV, antiretroviral therapy, attrition, faith healing, herbal remedies, Zambia

## Abstract

**Introduction:**

Despite the relatively effective roll-out of free life-prolonging antiretroviral therapy (ART) in public sector clinics in Zambia since 2005, and the proven efficacy of ART, some people living with HIV (PLHIV) are abandoning the treatment. Drawing on a wider ethnographic study in a predominantly low-income, high-density residential area of Lusaka, this paper reports the reasons why PLHIV opted to discontinue their HIV treatment.

**Methods:**

Opened-ended, in-depth interviews were held with PLHIV who had stopped ART (n = 25), ART clinic staff (n = 5), religious leaders (n = 5), herbal medicine providers (n = 5) and lay home-based caregivers (n = 5). In addition, participant observations were conducted in the study setting for 18 months. Interview data were analysed using open coding first, and then interpreted using latent content analysis. The presentation of the results is guided by a social-ecological framework.

**Findings:**

Patient attrition from ART care is influenced by an interplay of personal, social, health system and structural-level factors. While improved corporeal health, side effects and need for normalcy diminished motivation to continue with treatment, individuals also weighed the social and economic costs of continued uptake of treatment. Long waiting times for medical care and placing “defaulters” on intensive adherence counselling in the context of insecure labour conditions and livelihood constraints not only imposed opportunity costs which patients were not willing to forego, but also forced individuals to balance physical health with social integrity, which sometimes forced them to opt for faith healing and traditional medicine.

**Conclusions:**

Complex and dynamic interplay of personal, social, health system and structural-level factors coalesces to influence patient attrition from ART care. Consequently, while patient-centred interventions are required, efforts should be made to improve ART care by extending and establishing flexible ART clinic hours, improving patient-provider dialogue about treatment experiences and being mindful of the way intensive adherence counselling is being enforced. In the context of insecure labour conditions and fragile livelihoods, this would enable individuals to more easily balance time for treatment and their livelihoods. As a corollary, the perceived efficacy of alternative treatment and faith healing needs to be challenged through sensitizations targeting patients, religious leaders/faith healers and herbal medicine providers.

## Introduction

Even though the benefits of ART in reducing mortality among people living with HIV (PLHIV) are well documented [1–6], some PLHIV still drop out of treatment programmes [6,7]. An analysis of 33 patient cohort studies from 13 Africa countries revealed that on average, 60% of patients were retained in ART care after 2 years of initiation [8]; another study has reported retention rates of 75% at 12 months and 67% at 24 months [9]. Reasons for patient attrition from ART care have included the use of traditional medicine [10–12], costs, side effects and stigma [13], and belief in faith healing [12,14,15].

Zambia is one of the countries in sub-Saharan Africa (SSA) worst hit by the HIV pandemic. Since 1984 when the first case of HIV was detected, the country recorded a steady increase in HIV prevalence, peaking at around 16% in the 1990s before levelling off and marginally declining to current rates [16]. Recent estimates indicate that 14.3% of the Zambian population (aged 15 to 49 years) is living with HIV [17]. In 2005, the Zambian Government introduced free ART services in public sector health facilities. This resulted in a rapid increase in the number of PLHIV on treatment, from 30,103 at the end of 2005, to an estimated 283,863 (adults and children) at the end of 2009 [16].

Despite the provision of free treatment, some PLHIV still drop out of ART care, running the risk of developing drug resistance and negative health outcomes. Three case studies in Lusaka, Zambia, identified the use of herbal remedies and opting for faith healing as reasons for discontinuation of ART [18] while two other studies [19,20] reported concerns about side effects, food insecurity and mistrust of medication as some of the factors influencing patient attrition from ART care. While previous studies have provided insight into the various reasons for non-uptake of ART, there is dearth of evidence on how different factors interact to influence health-seeking behaviour, including patient-health care provider interaction. In this study, we explored the reasons for patient attrition from ART care in an urban community of Lusaka, Zambia, in order to understand how PLHIV balance their decisions based on the underlying framework of socio-economic, cultural and health-system-related factors.

## Methods

### Theoretical framework: social-ecological framework

**Figure 1:**
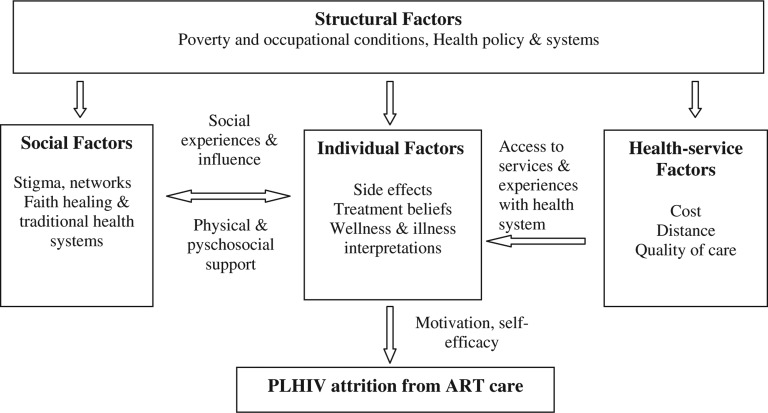
Social-ecological framework: adapted from Roura et al. (2009) with permission from Mary Ann Liebert, Inc. **Note:** Left-right arrow suggests a bi-directional relationship. For instance, failure to cope with side effects (individual level factor) may force PLHIV to opt for faith healing (social factor). Similarly, lack of social network support (social factor) can affect attitude towards treatment leading to low self-efficacy.

Over the years, various theories and models have been developed to help understand health-seeking behaviour [21,22]. We adapted the social-ecological framework by Roura *et al*. [23] to explore the factors that influenced patient attrition from ART care (Fig. 1). The theoretical framework provides a comprehensive approach to understanding health-seeking behaviour. It frames human behaviour as a function of personal and environmental (social, economic, political, health system) factors [21,23–25].

The approach avoids exclusive focus on either individual or environmental factors as separate analytical factors, but pays attention to the dynamic interaction of both personal and non-personal factors in understanding health-seeking behaviour [23,25]. The premise of the framework is that individuals’ health-seeking behaviour is located in social, institutional and physical environments, and consequently, behaviour shapes and is shaped by, the social environment [23,26]. Health-seeking behaviour is therefore construed not as something that exclusively resides in the individual, but rather as a reflection of wider interactive situational processes [22,23,27].

The social-ecological framework categorizes the factors influencing health-seeking behaviour into individual, social, health system and structural factors [23]. Individual-level factors comprise personal characteristics such as perceived disease severity, illness and wellness interpretations, and knowledge and attitudes towards treatment, as well as somatic response to medication [23,28].

Social factors include interpersonal relationships with marital partners, family members and peers that in turn affect individual behaviour and action [23]. Social factors also include relationships with, and influence of, social institutions like the church and traditional healthcare systems which are deeply embedded in people's socio-cultural systems. Morality, norms and values reflect and shape social processes. Social exclusion, discrimination and stigmatization are located on this level, too. Similarly, the relationship between health professionals and clients is structured by social order; social distance and poor relationship between health workers and patients affect access to treatment [28].

Structural factors are those external to the control of the individual [29,30]. These include endemic poverty, livelihood circumstances, health policies, laws and regulations, and the financing of the healthcare system, which is linked to the national economy [31,32].

Health system factors relate to the way health services are organized and delivered [29]. These include availability and accountability of services, attitude of providers, waiting time and distance to health facilities [23,29].

Because of its comprehensive and multi-faceted approach, the social-ecological framework avoids the theoretical divide between “individualist” and “structuralist” models by treating both paradigms as complementary and lying on the same continuum [23]. Individual, social, health system and structural-level factors are viewed not as mutually exclusive, but inextricably linked and in some cases mutually reinforcing. For instance, health service interventions such as reduced waiting times (health system factor) may improve patient adherence behaviour through motivation to seek treatment (individual factor); patients’ contact with the health system are likely to be influenced by their attitude and beliefs about treatment, and their lay interpretation of illness and wellness [29]. Sometimes a combination of different level-factors may influence non-uptake of treatment. For instance, pervasive stigma (social factor) and long waiting time at ART clinic (health system factor) may coalesce leading to lack of self-efficacy (individual factor) resulting in patient attrition from ART care.

### Research setting

The study was conducted in a predominantly low-income, high-density urban residential setting of Lusaka, Zambia. The setting comprises multilingual ethnic groups, with *Bemba* and *Nyanja* the most widely spoken local languages. The socio-economic status and housing conditions of the residents are mixed, but predominantly poor. The majority of the residents are either in the informal sector of the economy or in lowly paid jobs of the public and private sectors. A lot more other people are not in any form of employment.

Health services in the area are mainly provided by a public sector clinic which provides both in- and out-patient health services. The clinic serves an estimated catchment population of more than 150,000 people. The clinic has been providing ART since 2006 and by mid-2010, the clinic had over 5,000 people on ART, and over 5,000 registered for pre-ART. In addition to the requirement for every patient to have a treatment supporter, called “buddy”, the ART clinic runs a treatment support group, and employs paid lay treatment supporters that follow-up and trace treatment defaulters. A community-based home-based care (HBC) programme also provides physical, psychosocial and treatment support to PLHIV.

There are also a myriad of traditional healers in the area; some of them advertise their health services, including treatment for sexually transmitted diseases (STIs) and “immune boosters” for HIV. Religion, particularly Christianity, also plays a pivotal role in the lives of many people, providing spiritual, material and social support. The major Christian churches in the area are the United Church of Zambia (UCZ), Roman Catholic Church, New Apostolic Church and the Seventh-Day Adventist Church (SDA). There is also a growing movement of evangelical charismatic Pentecostal churches, some of which claim to provide faith healing for different health problems, including HIV.

### Research design and study participants

A descriptive qualitative study nested within a wider ethnographic study was conducted. The study sample comprised PLHIV (≥18 years old and residents of the study setting), classified as lost-to-follow-up (LTFU)/defaulters by a local public sector clinic for missing their scheduled pharmacy pick up for at least 6 months (180 days). The revised Zambian ART treatment guidelines have reduced the LTFU threshold to at least 60 days [33]. Our sample also comprised ART staff of the local public sector clinic, home-based care providers, herbal medicine providers and religious leaders (Pastors) based in the study setting.

### Participant recruitment strategy

All participants were recruited through a purposive sampling strategy. This approach was used in order to identify appropriate key informants (i.e. clinic staff involved in the delivery of ART; religious leaders involved in faith healing and herbal medicine providers). The sampling strategy also helped identify PLHIV with unique and diverse experiences related to discontinuation of treatment. PLHIV who had discontinued ART were first contacted and recruited through ART staff. From patient records, patient locator information was extracted by ART staff to identify eligible participants. Other research participants were identified and recruited through their home-based care providers. The ART staff and home-based caregivers explained the purpose of the research.

### Data collection and analysis

Data were collected between September 2010 and March 2011. Open-ended in-depth interviews were held with PLHIV who had stopped ART (n = 25), VCT/ART staff (n = 5), religious leaders (n = 5), herbal medicine providers (n = 5) and lay home-based caregivers (n = 5). The main research question posed to PLHIV was: “What made you decide to discontinue taking your HIV medication?” Key informants were asked the question: “Why do PLHIV started on treatment later discontinue their medication?” The majority of the interviews were recorded using a digital audio recorder. Where digital recording was not possible due to respondent refusal or malfunctioning of the digital recorder, the interviewer took detailed handwritten notes. Some of the interviews were conducted in the local language, *Nyanja*, and others in English. The interviews usually lasted between 30 and 45 minutes.

Structured observations were also conducted at the local public sector ART clinic, at religious gatherings, including church services. Social contact with residents, social institutions and the local health system provided more in-depth perspectives which helped situate PLHIV health-seeking behaviour into their lives and experiences. The observations also helped triangulate the data collected through in-depth interviews. All interviews and observations were conducted by the first author.

The interviews conducted in the local language were translated, and all interviews were transcribed verbatim. The interview transcripts were entered into, and managed using Atlas ti version 6 software. We used latent content analysis as described by Graneheim and Lundman [34]. The transcripts were read several times to develop a sense of the whole, and then conducted open coding of the data. The codes were then compared for similarities and differences, and then grouped into categories on a manifest level. Themes were then generated by interpreting the categories for their underlying meaning.

Observational notes were manually analysed to develop broad themes that best captured and typologised the study setting, and described the livelihood and treatment experiences of PLHIV. In our study, the framework was not used for thematic coding but served as a guide to present the results in a structured way.

### Protection of research participants

As a component of a bigger research project, “improving equity of access to HIV care and treatment in Zambia”, the study was approved by the ethics committee of the state of Basel (EKBB) and the University of Zambia Humanities and Social Sciences Research Ethics Committee. Clearance was also obtained from the Ministry of Health, at national and district levels. Only PLHIV that had been contacted by ART staff and home-based caregivers and agreed to be interviewed were recruited for interviews. To ensure privacy and confidentiality, the interviews took place in different settings, mainly neutral locations depending on the preferences of the respondents. Where appropriate, PLHIV were given lunch and/or transport reimbursements (of up to US$10). All research participants provided written informed consent.

## Results

We first present individual-level, social and structural-level factors emphasized by PLHIV in order to give an overview of their main concerns. This is followed by findings that specifically relate to patient-healthcare provider interaction, in which individual, social, and structural factors are mirrored as well.

### Profile of PLHAs who had discontinued ART

A total of 25 PLHIV who had discontinued their treatment were included in the analysis. The majority of the respondents (n = 17) were women, and nearly half (n = 11) were aged between 25 and 34 years. The oldest respondent was a widow aged 48 years. More than half (n = 15) of the PLHIV were in informal employment. In terms of treatment history, 8 of the 25 PLHIV had been on treatment for up to a year; and less than half (n = 10) abandoned medication within a year of starting treatment (Table 1).

**Table 1:** Characteristics of PLHIV who stopped treatment

Characteristic	Proportion of PLHIV
**Age (years)**	
18 to 24	5
25 to 34	11
35 to 44	3
>44	6
**Sex**	
Man	8
Woman	17
**Marital status**	
Single	4
Married	12
Divorced/separated	7
Widowed	2
**Source of livelihood**	
Formal employment	4
Informal employment	15
Not working/dependant	6
**Time on treatment**	
≤12 months	8
1 to 2 years	6
2 to 3 years	6
3 to 4 years	2
>4 years	3
**Duration not on treatment (up to time of interview)**	
6 to 12 months	10
1 to 2 years	8
2 to 3 years	5
3 to 4 years	2

### Reasons for patient attrition from ART care

#### Individual-level factors

*Side effects and treatment beliefs:* Treatment-related side effects were a recurrent theme for discontinuing treatment. The frequently mentioned side effects were severe stomach pain and diarrhoea, severe leg pain and headache, body rash, fatigue and vomiting. While PLHIV acknowledged that side effects were inevitable, they felt that the side effects were inimical to their health and comfort and interfered with their engagement in livelihood activities. An unemployed 33-year-old woman narrated how side effects sapped her energies and made her incapable of engaging in livelihood activities:

The time I was on treatment, I used to feel weak all the time. I used to feel dizzy. I used to fail to carry even a 10 litre container of water. I could not do any piece works to make money. Although I still cough, its much, much better now than when I was on treatment.

Some PLHIV expressed concerns about the dangers of treatment to their lives and long-term health. One respondent narrated: “*Those drugs almost killed me. I almost went mad. Today, if I was still taking those drugs, I would either be mad or I would have died by now*” (28-year-old woman). Although not explicitly expressed by others, one PLHIV felt that while the drugs generally improved health, they also inflicted debilitating effects in the long term:

The problem with ARVs is that you become fat first before you die, that you look healthy outwardly, but inside your body, you are rotten, being eaten up by the drugs. (48-year-old woman)

*Treatment fatigue and low self-efficacy:* While side effects were a reason for abandoning treatment, improving wellbeing decreased motivation to continue on treatment too. Often triggered by physical improvements of corporeal health, some PLHIV saw no need to continue with treatment. One participant said: *“To tell you the truth, I have consumed those drugs, I am now tired ….Now that I feel better, I decided to stop”* (48-year-old woman).

*Change of identity:* A few PLHIV emphasized their desire to feel “normal”: continued uptake of treatment often reminded them of being “sick”, having an incurable and fatal condition, and dependent on medication. Therefore to contruct a positive and “healthy” image of themselves, they opted to discontinue their medication. As one PLHIV explained:

Taking ARVs always reminds you of HIV. Sometimes you just want to forget about it, to be like others who are not infected. So when you feel ok, you want to stop taking the drugs so that you also feel that you are also a normal person. (34-year-old man)

#### Social-level factors

*Fear of losing social and emotional support due to anticipated stigma:* Some PLHIV were fearful of involuntary disclosure of their status, either to their marital partners or other social network members and facing the prospects of social exclusion. Especially women, social support was crucial because of their surbordinate socio-economic status. Some women were full-time housewives and/or small traders operating small makeshift shops locally called *tutembas*. Consequently, to avoid involuntary disclosure of their HIV status, they opted to stop treatment in order to preserve social support systems. One woman narrated her dilemma:

I feared that my husband would know, and if he knew, he was going to divorce me. Where can I go if I got divorced? Who will look after my children? I just said to myself that it is better I stop medication so that I can protect my marriage, and so that my children can have a future. (32-year-old woman living with HIV)

*Social network influence and experiences:* In some cases, PLHIV stopped treatment altogether or opted for faith healing and herbal remedies after being influenced by, and drawing on the health experiences of, other PLHIV who had stopped treatment. This was confirmed by ART staff, home-based caregivers and a few PLHIV. As an ART staff explained:

Some people boast [among their friends] that “here I am, I stopped medication but I am just fine”. So when other people see that, they also think that you can stop treatment and nothing would happen to you.

*Faith in God:* Personal relationship with, and trust in, a surpernatural being (God) also led to patient attrition from ART care. The majority of the respondents interviewed professed being Christians and some narrated how being “born-again” and their “personal relationship” with God dissuaded them from continuing with treatment. A 47-year-old widow who underwent some healing sessions explained:

I give praise to God that He healed me. I never thought that I would be healed because I was HIV positive. I used to take my medication every day, in the mornings and in the evenings. And at the clinic, they told me that if you stop taking your medication, you will die. But me, I stopped treatment and I have not died, I am still alive. God is great. Glory be to God.

Observations revealed that some local Pentecostal churches hosted healing sessions for people suffering from different ailments, including HIV. One Pentecostal church conducted healing prayer sessions every Saturday; on the wall of the local church read the banner: *“Come for counselling, deliverance and healing from all sickness and disease*”. Another Pentecostal church conducted “*deliverance and healing*” sessions every Wednesday and Thursday afternoon. During one of the church services, a middle-aged woman testified about how she was found HIV positive at a local public sector clinic and put on medication but opted to put her faith in God. She testified in church:

I embarked on prayer and fasting …. I told the devil that you are a liar. I asked God, can you show your power, and thank God, I am cured. Our living God is a God of wonders.

The practice of faith healing was confirmed by evangelical Pastors, ART staff and home-based care providers. People moved across Christian denominations in search of healing. An ART staff recounted an incident where ARVs were dumped at the ART clinic during the same week that a visiting Pastor conducted a series of “*deliverance and healing*” sermons in the area.

One of the local Pastors interviewed explained:

We pray for them and never lose hope even at the point of death. We still stand by the word of God, that God is able to cure or heal you. If God does not cure you, it is because of His personal reasons.

Another Pastor proclaimed:

The Bible teaches us that the things which are impossible with men are possible with God.

*Opting for alternative treatment:* In the study setting, alternative forms of treatment co-existed with ART care. PLHIV accessed herbal remedies either from traditional healers or from herbal medicine traders. Some of these remedies were being used either as purported cures or as “*immune boosters*”. These remedies included crocodile fats, *moringa oleifera* and *aloe vera* gel (also locally available in plant form called *tembusha*). While some PLHIV initially used herbal medicines concurrently with ART, overtime, they reported exclusively opting for herbal remedies. For some, opting for alternative treatment was triggered by ART-induced side effects, dissatisfaction with ART care and inability to stick to the stringent ART regimen. For other PLHIV, the quest to get cured attracted them to use herbal remedies. One PLHIV explained:

I never had any problem with the health workers. But I just decided on my own when I heard that crocodile fats cure, so I decided to start taking crocodile fats in the hope of being healed. (46-year-old man)

A herbal medicine trader supplying herbal medication to PLHIV claimed that her herbal remedies were as effective as ART in boosting the immune system:

I have different types of herbs which improves the health of people who have HIV. Some of the herbs boost the immune system. I also have other herbs that cleanse the body of toxins. Some of my customers were not recovering when they were taking ARVs,but when they started taking my herbs, their health improved.

#### Structural-level factors

*Insecure labour conditions:* For some PLHIV, the fear of losing their jobs on account of their HIV status, anticipating stigma, hindered them from accessing treatment. Most people, even when employed, did not have proper contracts which would protect them in case of illness as foreseen by the law. People with job insecurity who also reported earning meagre income did not want to lose their livelihoods on account of disclosure of their HIV status. The lack of both formal and strong informal social safety nets reinforced the need to preserve sources of socio-economic support. One woman said:

I lost my marriage when I told my husband my status ….So I did not tell my boss that I was HIV positive, so I feared that if I told her, she was going to fire me. So I decided to keep quiet so that I keep my job ….Instead, I decided to stop going to the clinic so that she does not know my status. (30-year-old divorced woman)

Poor labour conditions that did not respect employees’ right of access to healthcare also made it difficult for PLHIV to reconcile time for treatment and their livelihoods. The majority of our respondents were engaged in informal, low-income livelihoods (i.e. bus drivers, house maids, construction workers, petty traders) and getting time off-work presented enormous opportunity costs. Some PLHIV experienced worse dilemmas. A Zambian truck driver working for a South African-based transport company narrated his harrowing experience:

My drugs got finished when I was in South Africa. The problem was that when I told my boss that I want to go to Zambia for treatment, he told me that “if you decide to go, you should never come back for work”. So you know how hard jobs are to find. So I just decided to hang around so that I do not lose my job. (32-year-old truck driver)

Sometimes PLHIV “cooked-up” stories that would generate compassion from employers such as a child, wife or husband being sick at home or the need to attend a funeral of a close relative. Even where permission was obtained, PLHIV had to tread a thin line between accessing treatment and securing their jobs. The frequency of getting permission had to be minimal to avoid being misconstrued by employers as not being committed to work. One 30-year-old PLHIV who lost his job twice, first as a plumber and then as a minibus conductor, after frequently getting permission from work to seek treatment narrated his ordeal:

Your health is your health, and what they [employers] care about is their business, doing their work. Sometimes if you ask for permission frequently, they think that you are just giving excuses. So, they fire you and replace you with someone else. That is how I lost my jobs.

#### Health system-level factors

*Competing priorities and dissatisfaction with ART care*. When livelihood problems and low perceived quality of care coalesced, incentives to stay on treatment diminished. For some PLHIV, long waiting time and frequent trips to the clinic presented enormous opportunity costs which they were not willing to forego. This was especially the case for those who reported living “*hand-to-mouth*” livelihoods or were in insecure employment and therefore frequent trips to the clinic “*forced*” them to choose between treatment and their livelihoods. Relatedly, the long clinic appointments often lasting almost a whole day, non-availability of some drugs and laboratory test results, and perceived rudeness of some clinic staff frustrated and dissuaded other PLHIV from seeking ART care. A woman in an HIV concordant marriage narrated their disillusionment with ART care:

Sometimes, you go there, they collect your blood, but the next time you go to the clinic, they tell you that your results got lost and you should give fresh blood and come again after two weeks. That frustrates a lot of people.… One time, my husband's results and my results got lost twice. (33-year-old woman)

Dissatisfaction with the way clinic staff responded to patients’ concerns included the problem of side effects, too. Several PLHIV reported not going back to the clinic to report side effects; others indicated that whenever they did, ART staff often dismissively told them that “*you will be fine once your body gets used to the medication”* (32-year-old man). The unresolved concerns about side effects often triggered PLHIV to opt for faith healing and herbal remedies.

*Paradoxical impact of intensive adherence counselling*. In order to reduce non-adherence, clinic staff included the so-called “defaulters” in special counselling programmes. The compulsory participation in intensive adherence counselling offered by the ART clinic involved reverting “defaulters” to weekly doses of treatment in which they were asked to report to the clinic weekly for adherence counselling for at least a month. This exasperated the balancing act between treatment and livelihoods. Several PLHIV described this approach as insensitive and a threat to their livelihoods. Looking livid, one 30-year-old woman complained:

Do we only live to go to the clinic or we also have other things to do in life? If I keep on spending time at the clinic, then, how I am going to look after myself and my family? What will we be eating with my family? What time would I have to go and do my work to earn a living? This really annoyed me and I got fed up with the clinic and decided to stop my medication.

Another PLHIV, a second-hand clothes trader, opted to stop treatment after being put on intensive adherence counselling. She reportedly attended her mother's funeral outside Lusaka and missed her clinical appointment by four days because she stayed longer for the funeral than anticipated. When she returned to the clinic, she was classified as a “defaulter” and immediately put on intensive adherence counselling despite not intentionally missing her clinical appointment. She complained:

I went with my medication but because I stayed there [at the funeral] for a week, my medication got finished. But then they [ART staff] just said that we will give you drugs for one week and put you on intensive adherence counselling. The counsellor just said you patients are a problem, you just want to come to the clinic whenever you feel like. This put me off. It was like I was being punished for attending my mother's funeral. (21-year-old woman)

## Discussion

The results illustrate how individual-level, social and structural-level factors influencing why patients discontinued ART care in an urban community of Lusaka are equally characterizing the patient-provider interaction. Every person who stopped ART did this knowing that this was against the recommendations of the health system.

When looking at individual-level reasons given by PLHIV, treatment was often interrupted when side effects became too severe or were perceived as compromising quality of life, a finding reported in many other studies, too [35–38]. The health system's response that side effects would disappear after some time was not convincing to many PLHIV. Our findings suggest that the conception of being healthy is subjectively experiential rather than shaped by laboratory test results that cannot be felt [39,40]. Patient's perceptions of the impact of treatment on physical, physiological and psychological wellbeing influenced their decisions. For instance, lack of motivation to continue with treatment was influenced by perceived improvement of corporeal health. In this case, good health and wellbeing are not only about improved CD4 cell count and lower viral load as measured in bio-medical discourse, but also in terms of comfort and unfettered ability to participate in day-to-day life activities [40]. Indirectly, this shows that the expert opinions of the ART providers are not trusted enough to legitimate a continuation of treatment.

Individual health-seeking behaviour is also strongly influenced by, and responds to, factors in the immediate social environment [23]. Social exclusion and discrimination is anticipated in case of disclosure of one's positive status. PLHIV fear the breakdown of their marriages, families, and loss of employment. In the absence of institutional social safety net, individuals rely exclusively on social network support to cope with unforeseen events. However, when social support is threatened by involuntary disclosure of HIV status, individuals abandon treatment as a protective mechanism.

Public identity transformation was avoided by all means especially if PLHIV were dependent on their social support networks. A strong urge to be “normal” was characterized by a variety of strategies of PLHIV. One example are the religious coping strategies of PLHIV, which cannot be discussed separately from the stigma associated with the condition of being HIV positive.

Despite the availability of antiretrovirals and the good health of many PLHIV, the moral dimension of HIV has not diminished, at least in this community. Coupled with livelihood insecurity, disclosure of a positive HIV status breeds tremendous social insecurity and loss of material and emotional support by spouses, families and friends. PLHIV coping strategies, such as re-establishing normalcy through being healed, unfortunately often implies abandonment of antiretroviral therapy.

Structural factors influence patient attrition as well. Our findings further suggest that livelihood constraints characterized by low-income, labour condition constraints and absence of strong formal and informal socio-economic safety nets affected patient continuation with treatment. While previous studies have found no correlation between poverty and adherence to treatment [41,42], other findings have reported the negative impact of livelihood constraints on treatment adherence [43,44]. Our findings suggest that in settings with endemic poverty levels and weak social safety net, individuals are inclined to avoid actions that would expose them to further socio-economic vulnerability.

These findings need to be contextualized. Zambia ranks poorly (164 out of 187 countries) on the human development index (HDI) [45]; 68% of its population falls below the national poverty line [46]; and 90% of the labour force is in informal sector employment [47]. Consequently, even where PLHIV are motivated to continue accessing treatment, health-seeking behaviour is undermined by coalesced effect of non-individual level factors such as fragile livelihoods, health system factors like long waiting times for medical care, which in turn compels PLHIV to prioritize their livelihoods over their health. Thus, to still maintain “good” health, some PLHIV opt for alternative forms of healthcare, like herbal remedies because they are easily accessible and do not impose inordinate opportunity costs.

Since the beginning of ART care, the health sector has tried many support strategies to improve adherence. Nonetheless, there are shortcomings of ART delivery, which are partly due to resource constraints. Similar to findings from previous studies [48,49] long waiting times at health facilities affected patient access to treatment. In contrast, however, neither the costs of treatment nor distance to the medical facilities were influencing patient attrition from ART care because ART services were free and within reach in this urban community.

Unexpectedly, however, specific interventions aimed at improving adherence to ART seemed counter-productive. This was the case for putting “defaulters” on intensive adherence counselling. What was striking in our study, which previous studies in Zambia have not reported [19,20], was the negative impact of intensive adherence counselling on patient retention in ART care. For patients, this was interpreted as being “punished” for circumstances beyond their control. Consequently, while aimed at achieving positive “quantitative aspects of HIV management and monitoring” [40], intensive adherence counselling was also achieving the opposite. Our findings corroborate the findings of Tugenberg *et al*. [50] who found that heavy insistence on adherence by clinicians forced some PLHIV to abandon medical visits to avoid confrontations with their doctors for non-adherence. Thus, perceived unresponsiveness of the health system pushes patients to seek alternative forms of healthcare, such as faith healing [15,51,52] and traditional medicine [12,18,53].

### Possible limitations of the study

This study was conducted in an high-density, generally poor urban setting, with a small purposively chosen sample of PLHIV. The findings may not be fully generalizable to other settings. Each setting may have unique characteristics that may influence health-seeking behaviour. However, our study has generated new insights about patient-provider interactions, the role of faith healing and traditional medicine, which may be generalizable beyond our setting and which contributes to the body of evidence on factors influencing patient retention in ART care.

## Conclusions

Patient attrition from ART care is not exclusively an individual choice, but affected by a complex and dynamic interplay of personal, social, health system and structural-level factors. Even for the most motivated patient, being on life-long treatment is not easy. Patients have to balance the exigencies of treatment with the effects of accessing treatment on their social, physical, mental and economic well-being. Therefore, while patient-centred interventions are critical to promote adherence, there is need to be mindful of, and address the influence of, non-personal-level factors which interact to influence health-seeking behaviour. This is crucial to achieve needs-based, demand-driven and beneficiary responsive ART care and ultimately retain patients in treatment programmes.
