# Corrigendum for Individual and contextual factors influencing patient attrition from antiretroviral therapy care in an urban community of Lusaka, Zambia

**DOI:** 10.7448/IAS.16.1.18590

**Published:** 2013-02-28

Regarding the paper ‘Individual and contextual factors influencing patient attrition from antiretroviral therapy care in an urban community of Lusaka, Zambia’ by Maurice Musheke, Virginia Bond and Sonja Merten

Published in *Journal of the International AIDS Society Supplement 1*. Citation: http://www.jiasociety.org/index.php/jias/article/view/17366 ∣ http://dx.doi.org/10.7448/IAS.15.3.17366

The categories in Table 1 are not clear. The correct table is displayed below

**Table 1:** Characteristics of PLHIV who stopped treatment

Characteristic (n=25)	Number of PLHIV
**Age**	
18–24 years	5
25–34 years	11
35–44 years	3
>44 years	6
**Sex**	
Male	8
Female	17
**Marital status**	
Single	4
Married	12
Divorced/separated	7
Widowed	2
**Source of livelihood**	
Formal employment	4
Informal employment	15
Not working/dependant	6
**Duration on treatment**	
<12 months	8
1–<2 years	6
2–<3 years	6
3–≤4 years	2
>4 years	3
**Duration not on treatment (up to time of interview)**	
6–<12 months	10
1–<2 years	8
2–<3 years	5
3–<4 years	2

